# Translation from the German

**Published:** 1872-06

**Authors:** Ch. Raushenberg

**Affiliations:** Atlanta, Georgia


### TRANSLATION FROM THE GERMAN.

BY CH. RAUSHENBERG, M.D., ATLANTA, GEORGIA.

*ON THE TENOTOMY OF THE TENSOR TYMPANI MUSCLE. By Dr. A. W. Calhoun, Georgia. From the Aural Clinic of Dr. Weber, Berlin.*

The operation of the tenotomy of the tensor tympani, lately introduced into aural medicine by Dr. F. E. Weber, must, undoubtedly, be considered an eminent progress in the modern therapeutics of that branch of medicine. This is not my opinion alone, but that of every one who has had the opportunity to witness this operation, with its astonishing results; and Prof. Gruber, of Vienna, has lately expressed himself in the same sense, in a lecture delivered on the same subject before the Society of Physicians of Vienna.

As, however, everything which so far has been said on this subject is contained alone in the special organ for aural diseases—*Monatsschrift fuer Ohrenheilkunde*—it will be of interest to the readers of the *Deutsche Klinik* to hear on this subject from a third person, who is engaged in otiatrics, and has several times been in the situation to witness this operation, and its consequences, in the clinic and private practice of Dr. Weber. This will best be accomplished by giving the history of a case which has been several months under my own observation. I presented, previously, a few preliminary remarks about the intention of the operation.

The tensor tympani presides, as is well known, over the changes in the tension of the tympanum. Late investigations have further established, that the tension of the ossicula, and the more or less intimate contact between the foot-plate of the styrup (basis stapis) and the labyrinth, depend in the same manner on the tension of this muscle. Thus, in consequence of its elastic tension, the labyrinth, even under normal conditions, continues under a permanent pressure from the cavity of the tympanum. Whenever this tension slackens, whenever this muscle is divided, the traction, which draws the ossicula and the styrup amongst them in the direction of the labyrinth, ceases, and the transmission of the sound-waves to the labyrinth becomes weaker and less direct. To the contrary, when the muscle is diseased, when it is in a state of spasmodic or permanent contraction, with its tendon retracted, the styrup must be pressed further into the labyrinth, and an increase of pressure within the same must result. If this is moderate, it manifests itself functionally by moderate subjective sounds. In proportion to the increase and duration of this intra-labyrinthic pressure, circulatory obstructions and their consequences must develop themselves within the same, and the oscillatory faculty of the intra-labyrinthic formations must decrease, and symptoms of vertigo, and particularly constantly increasing difficulty of hearing, must attach themselves to the more and more increasing subjective sounds. The excessive tension of the tympanum and of the chain of the ossicula, and a deviation of their normal position from each other in consequence of the morbid contraction of this muscle, must also receive a due share of consideration in the explanation of these phenomena.

I abstain from the discussion of other secondary tympanal changes, and will only remark, that as these conditions can be deduced from the phantom and from preparations, and their existence demonstrated by dissection of persons who were suffering from difficulty of hearing, the positive diagnosis of their existence can also be made, in many cases, on living individuals.

The tenotomy of the tensor tympani is performed for the purpose of abolishing abnormal intra-tympanic and intra-labyrinthic pressure, as far as it depends upon a retraction of the tensor typani; still, it must afford chances of relief even where an increase of intra-labyrinthic pressure and a subsequent suffering of the acoustic nerve exists from other causes, because it abolishes even the normal pressure of the styrup on the labyrinth.

The most interesting and most striking case which I have observed, of the tenotomy of the tensor tympani, is the following:

Miss Straubel, Rentière at Berlin, living 179 Schoenhauser Avenue, a very intelligent lady of middle age, applied for treatment in December, 1871. Complains of unceasing noises in the ears, which have existed about five years constantly, and have, particularly of late, steadily increased to such an extent, that they deprive the patient of her rest at night. Marked vertigo, a high degree of difficulty of hearing principally in the right, the prominently diseased ear, existed at the same time. When the patient participated in a conversation with several persons, the impressions of sound became so much intermingled to her, that she could only understand single sentences; in conversation with one person, slow and distinct articulation was necessary to make a full understanding possible. When the left ear of the patient was tightly closed, she could hear, but not understand, what was spoken behind her at a distance of one foot. The test with the box-watch, which, under normal conditions, can be heard about forty feet, furnished relatively favorable results: its ticking could be heard on the right side, a distance of one foot; on the left side, from twelve to sixteen feet.

Under continued examination, the patient, in consequence of increased exertion of the ear, could finally hear only about half the distance, while the noises in her ears increased during these exertions. The subjective aural sensations were compared to the sound of water boiling in a tea-kettle, intermixed with pulsation sounds; they changed very much in intensity, and became almost unbearable when the patient felt very much relaxed.

Both membranæ tympani presented the image of intense retraction and hypertension, with axillar rotation of the malleus. These changes were more marked on the right side, where the tympanum appeared drawn back, so as to present an umbilical depression, and the processus brevis of the malleus caused a sharp-edged protrusion on the tympanum; the cone of light was perceptibly indicated, and turbidity of the edges of the tympanum existed only to a very small degree, and the gloss of the tympanum was not changed.

On longer examination, however, marked injection of the vessels of the malleus became visible, principally while rarefaction of the air in the external meatus was produced by the pneumatic funnel. As long as, by this process, the tympanum was forcibly drawn outwardly, the patient experienced a very marked temporary relief. This was interpreted as an evidence of a counteraction of the abnormal contraction of the tensor tympani by the aspiration, which produced an outward traction of the tympanum.

An examination of the pharynx did not show any catarrhal phenomena, while the uvula appeared somewhat thickened, and was hanging toward the right side. The Eustachian tubes were easily passable by bougies, as well as the air-douche.

As the patient appeared anæmic and very much weakened, she was put on a tonic treatment. Different preparations of iron were administered for about one month; her general health improved, but no change was perceptible in the disease of the ear. Several local remedies—air-douche, section of the posterior tympanic fold, electricity—did not establish permanent improvement.

As the patient desired to be relieved, at any price, from the horrible suffering produced by the vertigo and the sounds in her ears, even if her faculty of hearing should be impeded still more, the operation of tenotomy, on the basis of a minute differential diagnosis and detailed consideration of the etiology of the case, was performed by Dr. Weber, on the 12th of January, 1872, in my presence.

The operation succeeded well, and the different acts of it were observed by me, with the aid of the aural speculum attached to the head-holder. The pain was pronounced to be inconsiderable. I saw the hook-knife introduced through the small wound of the tympanum, into the cavity of the latter, in such a manner as to occupy the space above the tendon of the tensor tympani, and heard a screaking noise at the moment the section was made; the hemorrhage was very small, and the pale mucous membrane of the tympanic cavity could be seen through the wound of the tympanum, stretched open to a considerable gap. Immediately after the operation, no improvement of hearing was perceptible; the patient felt weak and much excited, and asserted that she suffered from severer pulsations in the ear. The same is stopped up tightly, and oleum terebinthinæ administered—a remedy which seems to have a prophylactic value against inflammations of the ear.

January 13, 1872.—Patient states that she has had no pains in the ear. Tympanum very slightly injected; edges of the wound covered with coagulated blood; walls of the tympanic cavity pale, and not injected; hearing distance for the box-watch not improved—it is exactly one foot; after using the air-press, the hearing distance increased to two and a half feet. Patient asserts most positively that all the ear-sounds, which she used to compare with the hissing noise of boiling water, can not be heard any more, even by the closest attention, while the sensations of pulsation in the ear have remained unchanged, but can be momentarily suspended by compression of the carotis of the same side.

January 15, 1872.—Hearing distance only eight inches; after the air-douche, two feet. Patient feels much freer in her head; the hissing sounds remain gone; pulsations still present.

January 16, 1872.—Slight hissing sounds have reappeared since yesterday evening, in conjunction with slight coryza and slight swelling of the tuba Eustachii. Patient feels free in the head—has no vertigo; hearing distance increased from three to four feet. No sign of reactive inflammation about the tympanum; the edges of the operation-wound slightly glued over with bloody substances, but diminished to half its size. A strong rarefaction of the air in the external meatus, by the pneumatic funnel, produces heavy flapping motions of the tympanum—a proof that tne counter-tension of the tensor tympani has been abolished, and that this muscle has been divided. Patient feels no inconsiderable pain during this process of rarefaction, and her ear feels, according to her own language, "as if everything was about to be torn out." The tympanum has become reddened, and a small hemorrhage from the operation-wound is perceptible. Patient affirms that her ear feels better than it has for a long time. Hearing distance increased to six feet.

January 18, 1872.—Status idem.

January 19, 1872.—Hearing distance four feet; hissing sounds gone; tympanic wound glued up. Hearing distance after air-douche, six feet.

January 21, 1872.—Tympanum shows a reddish transparency. Patient describes a sensation as if a foreign body were in her ear; the existence of an increased secretion in the ear is suspicioned, and a sero-purulent fluid is removed from the tympanic cavity by Weber's catheter. Hearing distance increased to eight feet.

January 22, 1872.—Patient declares that she feels better than she has in two years. Head free; the noises heretofore heard in the sound ear have also disappeared. Hearing distance five feet. The tympanum shows again to to-day a very fine aperture within the cicatrix, as if it had only been imperfectly glued up before, and a perforation sound is perceptible during the air-douche.

January 23, 1872.—Hearing distance four feet; noises in the ears, and vertigo, still gone.

January, 29.—Status idem. The tympanic wound positively healed up; the air-douche and the aspiration cause flapping motion of the tympanum. The ear remained, from now on, almost uniformly at the same degree of improvement. The vertigo and the ear-noises remained entirely gone; only now then, after excitement, pulsations occurred. The faculty of hearing language had improved so extraordinarily that a loud conversation across three rooms could be carried on with the patient, and she could even follow a whispering conversation of several persons in a perfectly normal manner. If the left ear was closed up, she could only converse across one room, and heard the ticking of the box-watch a much greater distance than before the operation (three feet.)

If it is already to be considered an extraordinary success, that the phenomena of vertigo and ear-sounds were positively abolished, and the faculty of hearing very materially improved by the tenotomy performed in the right ear, it is certainly not less worthy of note, that the less developed morbid phenomena of the left ear were also made to disappear entirely.

Dr. Weber believes that he must explain such favorable effects on the non-operated ear as the result of a liberation of the central extremities of the acoustic nerve from the continued transmission of the intra-labyrinthic pressure irritation, which has been abolished by the operation on the effected side—thus also relieving the acoustic nerve of the sound side, and enabling it to return to a normal performance of its functions. Similar phenomena are observed in some cases of glaucoma, after the successful performance of iridectomy.—*Deutsche Klinik, No.* 9, 1872.

